# DNA damage and S phase-dependent E2F1 stabilization requires the cIAP1 E3-ubiquitin ligase and is associated with K63-poly-ubiquitination on lysine 161/164 residues

**DOI:** 10.1038/cddis.2017.222

**Published:** 2017-05-25

**Authors:** Valérie Glorian, Jennifer Allègre, Jean Berthelet, Baptiste Dumetier, Pierre-Marie Boutanquoi, Nathalie Droin, Cémile Kayaci, Jessy Cartier, Simon Gemble, Guillaume Marcion, Daniel Gonzalez, Romain Boidot, Carmen Garrido, Olivier Michaud, Eric Solary, Laurence Dubrez

**Affiliations:** 1 Université de Bourgogne Franche-Comté, LNC UMR1231, Dijon, France; 2 Institut National de la Santé et de la Recherche Médicale (Inserm), LNC UMR1231, Dijon, France; 3 Inserm U1170, Gustave Roussy, Villejuif, France; 4 Centre Georges-François Leclerc, Dijon, France; 5 Université Paris-Sud, Faculté de Médecine, Le Kremlin Bicêtre, France

## Abstract

The E2F transcription factor 1 is subtly regulated along the cell cycle progression and in response to DNA damage by post-translational modifications. Here, we demonstrated that the E3-ubiquitin ligase cellular inhibitor of apoptosis 1 (cIAP1) increases E2F1 K63-poly-ubiquitination on the lysine residue 161/164 cluster, which is associated with the transcriptional factor stability and activity. Mutation of these lysine residues completely abrogates the binding of E2F1 to *CCNE*, *TP73* and *APAF1* promoters, thus inhibiting transcriptional activation of these genes and E2F1-mediated cell proliferation control. Importantly, E2F1 stabilization in response to etoposide-induced DNA damage or during the S phase of cell cycle, as revealed by cyclin A silencing, is associated with K63-poly-ubiquitinylation of E2F1 on lysine 161/164 residues and involves cIAP1. Our results reveal an additional level of regulation of the stability and the activity of E2F1 by a non-degradative K63-poly-ubiquitination and uncover a novel function for the E3-ubiquitin ligase cIAP1.

The E2 factor (E2F) protein family consists of eight transcription factors (E2F1–8) that are essential regulators of cell proliferation, cell differentiation, DNA damage response, cell death and cell senescence. Their foremost functions are in cell cycle regulation as they participate in the dynamic expression of molecules driving cell cycle progression. E2F1 is the most extensively studied member of this family. Its protein expression level is increased in many human cancers such as hepatocarcinomas, digestive adenocarcinomas and ovarian cancers, and its downregulation in p53- and RB-compromised cancer cells can trigger their senescence.^1^ Further understanding of E2F1 expression regulation would help to develop strategies targeting this protein in cancer cells.

The expression and activity of E2F1 are regulated through post-translational modifications and association with different regulatory factors. E2F1 transcriptional activity is repressed by binding to the hypo-phosphorylated form of the pocket protein pRb (retinoblastoma protein).^2^ In the late G1 phase of cell cycle, the phosphorylation of Rb by cyclin/cdk complexes releases E2F1, allowing the transcription transcription of genes required for G1-S phase transition and DNA replication.^2^ E2F1 is subsequently degraded in late S and G2 phases by the ubiquitin proteasome system (UPS).^3^ A genotoxic stress can also induce the stabilization and activation of E2F1 that, in this situation, promotes cell death rather than cell proliferation.^4^ The turnover and activity of E2F1 are regulated by a number of post-translational modifications including phosphorylation, acetylation, methylation, neddylation, sumoylation and ubiquitination.^2, 4^

Ubiquitination consists of a multistep enzymatic process, the aim of which is to conjugate ubiquitin molecules on a lysine of target proteins. This process is mediated by a cascade reaction involving an ubiquitin-activating enzyme (E1), an ubiquitin-conjugating enzyme (E2) and an E3-ubiquitin ligase enzyme or E3-ubiquitin ligase enzymatic complex. The free N-terminus of ubiquitin or each of its seven lysine residues can act as an acceptor site for ubiquitin. At least eight types of ubiquitin chains can be formed depending on the ubiquitin acceptor residue. The nature of the ubiquitin chain defines the functional consequences of target protein ubiquitination. For example, Lys48 (K48) or Lys11 (K11)-linked chains promote the protein degradation by the proteasome, whereas Lys63 (K63)-assembled chains favor the protein recruitment into signaling platforms.^5^ E2F1 is ubiquitinated by the SCF^skp2^ (Skp2 (S phase kinase binding protein 2)-CDC53 (Cullin)-F-box) E3 ligase complex in the late S and G2 phase of cell cycle^6^ and by APC/C^cdc20^ (anaphase-promoting complex/cyclosome) in early mitosis.^7^ In addition, in early G1, the APC/C^cdh1^ E3 ligase complex promotes the conjugation of the K11-linked ubiquitin chain on E2F1.^8^ In all these situations, ubiquitinated E2F1 is delivered to proteasome for degradation. A K63 ubiquitination of E2F1 has also been reported^9, 10^ but the involved E3-ubiquitine ligase enzyme and the consequences of this modification remain unexplored.

Cellular inhibitor of apoptosis 1 (cIAP1) is a RING-containing E3-ubiquitin ligase of the IAP family. Thanks to its ability to induce the ubiquitination of signaling molecules, cIAP1 is a very potent regulator of the tumor necrosis factor receptor (TNFR) superfamily and NF-*κ*B transcription factor-activating signaling pathways.^11^ Therefore, cIAP1 is an important regulator of immune and inflammatory responses. In undifferentiated healthy cells and in some tumor cells, cIAP1 is expressed in the cell nucleus^12, 13, 14, 15^ where it interacts with E2F1 protein.^12^ In this study, we demonstrate that cIAP1 induces an accumulation of E2F1 modified through K63 ubiquitination on the lysine cluster 161/164, which is preceded by an arginine methylation step. The mutation of both lysine residues completely abolishes the transcriptional activity of E2F1 and its recruitment onto target gene promoters. A cIAP1-dependent accumulation of K63-ubiquitinated E2F1 is observed in the S phase of the cell cycle, when E2F1 activity is maximal and is required for the stabilization and the activation of E2F1 upon genotoxic stress.

## Results

### cIAP1 increases E2F1 protein expression and ubiquitination in an E3 ligase-dependent manner

**Figure 1:**
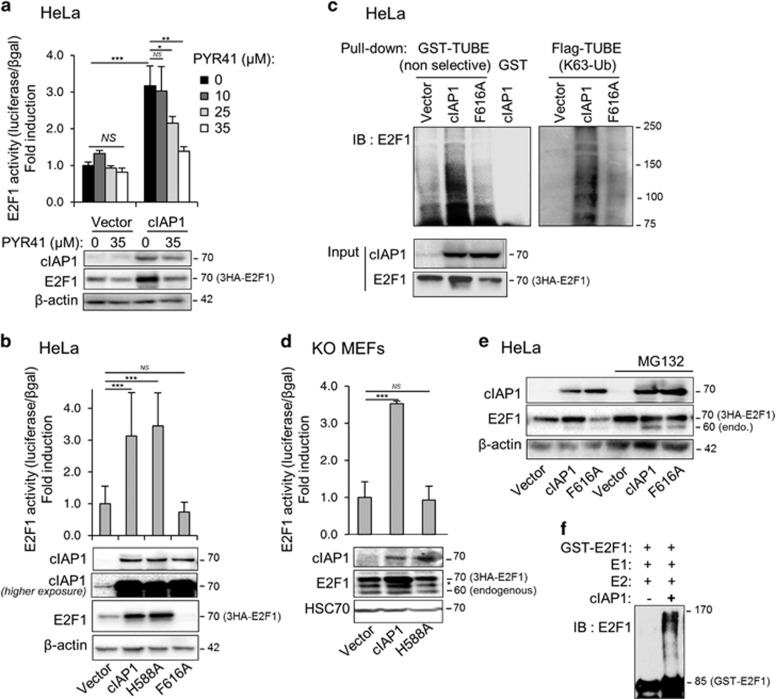
cIAP1 stabilizes and stimulates E2F1 in an E3-ubiquitin ligase activity-dependent manner. (**a, b** and **d**) E2F1 activity and expression in Hela cells (**a** and **b**) or in cIAP1^−/−^/cIAP2^−/−^ MEFs (**d**) transfected with CCNE promoter-Firefly luciferase reporter plasmid, pCMV-3HA-E2F1, along with empty vector (Vector), cIAP1, cIAP1-H588A (devoid of E3-ubiquitin ligase activity) or cIAP1-F616A (lacking dimerization capacity) mutant encoding vector. HeLa cells were treated for 24 h with PYR-41 10–35 *μ*M before analysis (**a**). Upper panels: E2F1 transcriptional activity was assessed in gene luciferase experiments. Luciferase activity was normalized to *β*-galactosidase activity and expressed as fold induction of promoter stimulated by E2F1 alone. Mean±S.D. of at least three independent experiments. Statistical analysis performed using Student’s *t*-test. ***P<0.001, **0.001<*P*<0.01, *0.01<*P*<0.1, NS *P*>0.1. Lower panels: the expression of the constructs was analyzed by a western blot analysis. *β*-Actin or HSC70 are used as loading control. *Unspecific bands. (**c**) Ubiquitination assay performed in HeLa cells transfected with 3HA-E2F1, His-Ubiquitin encoding vectors, with an empty vector, cIAP1 or cIAP1 F616A (F/A) mutant constructs. Ubiquitinated proteins were pulled-down by using non-selective or K63-TUBEs and ubiquitinated E2F1 is revealed by using an anti-E2F1 antibody. (**e**) Western blot analysis of cIAP1 or 3HA-E2F1 expression in HeLa cells transfected with pCMV-3HA-E2F1 and with empty vector (Vector), cIAP1 or cIAP1-F616A mutant (dimerization defective mutant) encoding vector, treated or not with MG132 5 *μ*M overnight. (**f**) *In vitro* ubiquitination assay of GST-E2F1 fusion protein immobilized on gluthatione sepharose and incubated with ubiquitin, E1 and E2 recombinant proteins with or without recombinant cIAP1

We previously demonstrated that cIAP1 is able to interact with E2F1 transcription factor and to increase its transcriptional activity.^12^ Given that cIAP1 is an E3-ubiquitin ligase, we assessed the role of ubiquitination reaction by using a pan-E1-ubiquitin enzyme inhibitor PYR-41. PYR-41 repressed, in a dose-dependent manner, cIAP1-mediated E2F1 transcriptional activation as analyzed using a cycline E gene reporter assay (Figure 1a). This E2F1 transcriptional activation is associated with a drastic increase in E2F1 expression level (Figures 1a, b and d), as well as an increase in E2F1 poly-ubiquitination (Figure 1c) as determined by pulling-down ubiquitinated proteins using tandem ubiquitin-binding entities (TUBEs). As non-degradative ubiquitination likely involves K63-linkage poly-ubiquitin chain, we confirmed our results by pulling-down ubiquitinated proteins using K63-linkage-specific TUBEs (Figure 1c). Importantly, these properties are lost when the cIAP1 F616A mutation prevents the formation of IAP dimers (Figures 1b and c). Consistent with the necessity of cIAP1 dimerization for its E3-ubiquitine ligase activity,^16^ the expression of the E3-ubiquitin ligase-deficient cIAP1 (H588A) conserved its capacity to stimulate E2F1 transcriptional activity in cIAP1-expressing HeLa cells (Figure 1b) but not in cIAP-deficient murine embryonic fibroblasts (Figure 1d). This suggest that, in HeLa cells, the E3 ligase defective H588A cIAP1 is likely to dimerize with the endogenous cIAP1 to enhance E2F1 protein level and to stimulate its activity in an E3 ligase-dependent manner. Interestingly, the expression of cIAP1 F616A induces a weak decrease in E2F1 expression (Figures 1b, c and e). Treatment of cells with the proteasome inhibitor MG132 stabilizes the expression of both E2F1 and cIAP1 and completely masks the effect of wt and the F616A cIAP1 on E2F1 expression (Figure 1e), suggesting a stabilization of E2F1 by cIAP1 and a proteasome-mediated degradation of E2F1 in the presence of the cIAP1-F616A mutant that could acts as a dominant-negative form. Finally, using an *in vitro* ubiquitin assay, we could demonstrate that E2F1 is indeed a substrate of the E3-ubiquitine ligase cIAP1 (Figure 1f).

### The cIAP1-mediated activation of E2F1 is associated with K63-poly-ubiquitinating on clustered lysines 161 and 164

**Figure 2:**
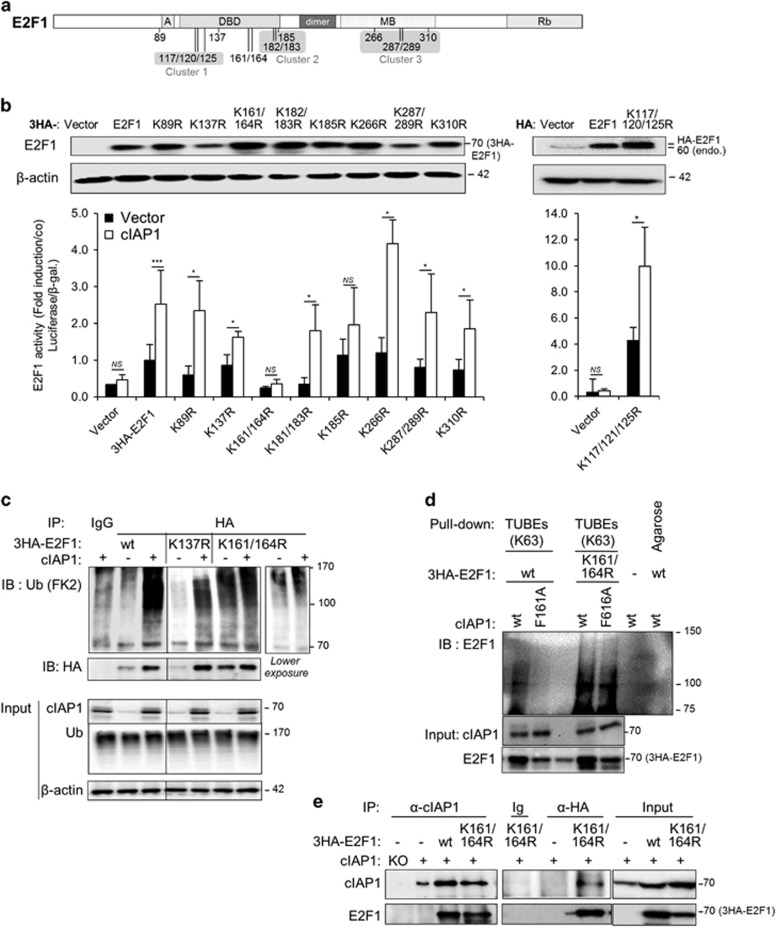
cIAP1 overexpression revealed a K63 ubiquitination of E2F1 on lysine residue 161/164. (**a**) Schematic representation of E2F1 protein structure indicating the lysine residues and lysine clusters. (**a**) Cyclin A: CDK2-binding domain; DBD: DNA-binding domain, dimer: dimerization domain; MB: marked box: Rb: Rb-binding domain. (**b**) Gene luciferase experiments performed in HeLa cells transfected with CCNE promoter-Firefly luciferase reporter plasmid, pCMV-HA (vector), HA-tagged-E2F1 (one single or 3HA) or HA-E2F1 mutants in which indicated K have been mutated into R along with empty vector (vector) or cIAP1-encoding vector. Luciferase activity was normalized to *β*-galactosidase activity and expressed as fold induction of promoter stimulated by E2F1. Mean±S.D. of at least three independent experiments. Statistical analysis performed using Student’s *t* test. ***P<0.001, *0.01<*P*<0.1, NS *P*>0.1. The expression of the E2F1 constructs was checked by a western blot analysis (upper panel). *β*-Actin is used as loading control. (**c** and **d**) Ubiquitination assay performed in HeLa cells transfected with 3HA-E2F1 wt, K137R mutant or K161/164R mutant, His-Ubiquitin, with an empty vector or cIAP1 or cIAP1 F616A (F/A) mutant constructs. E2F1 is IP by using specific anti-E2F1 antibody and ubiquitination is revealed by using pan-ubiquitin antibody (FK2) (**c**), or ubiquitinated proteins were pulled-down by using K63-specific TUBEs and ubiquitinated E2F1 is revealed by using an anti-E2F1 antibody (**d**). (**e**) IP analysis of the interaction of cIAP1 with E2F1 wt or K161/164 R mutant. Hela cells were transfected with 3HA-E2F1 encoding constructs and cIAP1. cIAP1 or E2F1 were IP using anti-cIAP1 or anti-HA antibody and cIAP1-E2F1 interaction was revealed by a western blot analysis. KO: cell lysate from cIAP1^−/−^ KO MEFs was used to check nonspecific reactivity of the antibody

The E2F1 protein contains 14 lysine residues, which are all potential acceptors for ubiquitin chains (Figure 2a). We constructed E2F1 mutants in which each lysine residue was replaced by an arginine residue and analyzed the capacity of cIAP1 to stimulate the transcriptional activity of these E2F1 mutants using a gene reporter assay (Figure 2b). Whereas the steady-state level of these mutants was different, no correlation was observed between the level of expression of E2F1 and its transcriptional activity (Figure 2b). As already reported,^17^ mutation of the lysine cluster 117/120/125, which is an acetylation site, strongly increased E2F1 transactivation activity (Figure 2b). The ability of cIAP1 to promote wt E2F1 transcriptional activity was retained by all E2F1 mutants, except the K161R/164R mutant for which cIAP1 was completely inactive and the K185R whose basal transactivation activity was weakly enhanced by cIAP1 (Figure 2b). An analysis of the ubiquitin profile of E2F1 in HeLa cells revealed that cIAP1 promoted the accumulation of the poly-ubiquitinated forms of E2F1 wt and K137R mutant, but it failed to increase the poly-ubiquitination profile of the K161R/K164R E2F1 mutant (Figures 2c and d), although it can still bind the protein, as demonstrated by co-immunoprecipitation (Figure 2e). Curiously, the K161/164R E2F1 mutant was strongly expressed and ubiquitinated at the basal state (Figures 2c and d). Finally, cIAP1 bound and promoted the accumulation of the ubiquitinated K185R E2F1 mutant as efficiently as the E2F1 wt (Supplementary Figure 1). Altogether, our results suggested that the cIAP1-mediated E2F1 transcriptional activity required ubiquitination on clustered lysine residues 161/164, located within the DNA-binding domain.

### The E2F1 lysine cluster 161/164 is required for E2F1 transcriptional activity

**Figure 3:**
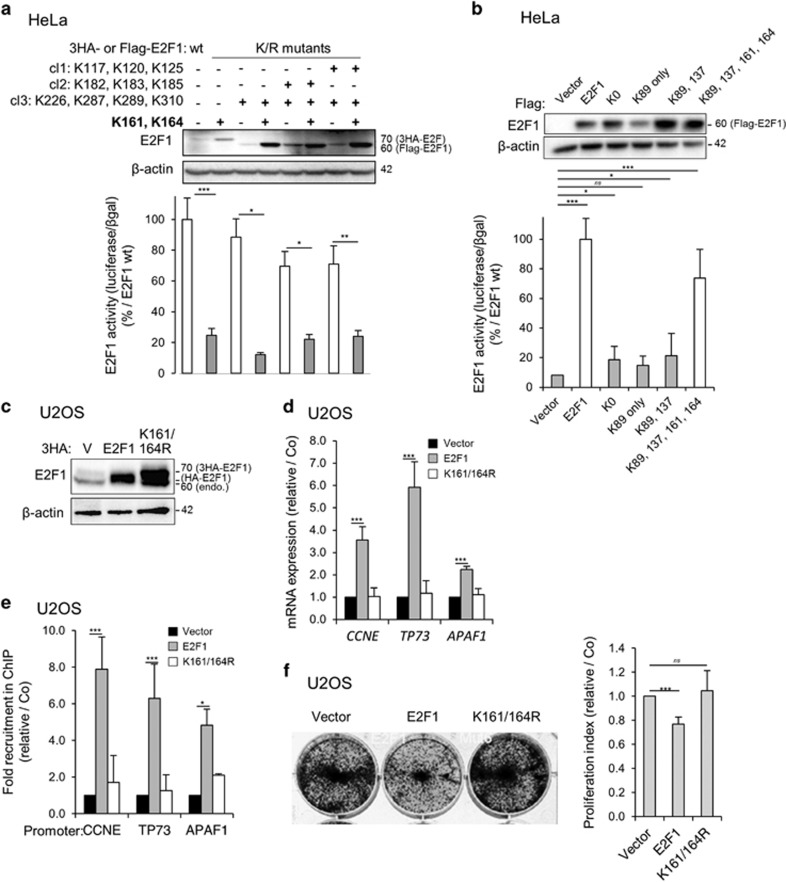
Cluster lysine 161 and 164 are important for E2F1 activity. (**a** and **b**) Gene luciferase experiments performed in Hela cells transfected with CCNE promoter-Firefly luciferase reporter plasmid and pCMV-3HA-E2F1 wt or E2F1 mutants constructs in which indicated K have been mutated into R (**a**) or (**b**) K0 (=mutation of every K into R), K89 only (=mutation of every K into R except K89), K89, 137 (=mutation of every K into R except K89 and K137) or K89, 137, 161, 164 (=mutation of every K into R except K89, K137, K161, K164). Luciferase activity was normalized to *β*-galactosidase activity. Results are expressed as % of activity measured with E2F1 wt. Mean±S.D. of at least three independent experiments. Statistical analysis performed using Student’s *t*-test. ***P<0.001, **0.001<*P*<0.01, *0.01<*P*<0.1, NS *P*>0.1. The expression of the constructs was checked by a western blot analysis (upper panel). *β*-Actin was used as loading control. (**c-f)** U2OS cells were transfected for 48 h with empty pCMV-3HA vector (V), pCMV-HA-E2F1 or pCM-3HA-E2F1 K161/164R mutant. (**c**) Western blot analysis of E2F1, 48 h after transfection. *β*-Actin was used as loading control. (**d**) Relative expression of *ccne*, *tp73* and *apaf1* mRNA as measured by RT-qPCR 48 h after transfection. Results were normalized to *hprt* mRNA and expressed relative to cells transfected with empty vector. Mean±S.D. of three independent experiments. (**e**) Chromatin IP experiments were performed using an anti-E2F1 or an irrelevant antibody (IgG). The levels of E2F1-associated genomic DNA region encompassing E2F1 binding site of *Cyclin E* (*CCNE*), *TP73* or APAF1 promoter were quantified by qPCR. Results were normalized to input and the recruitment with irrelevant antibody, and expressed as relative recruitment compared with cells transfected with empty vector. (**f**) Proliferation of U2OS cells transfected for 48H with empty vector, E2F1 or E2F1 K161/164R mutant, as analyzed by crystal violet staining. Mean±S.D. of three independent experiments. Statistical analysis performed using Student’s *t*-test. ***P<0.001, **0.001<*P*<0.01, *0.01<*P*<0.1, NS *P*>0.1

Mutations of the lysine residues of the cluster 1 (cl1: K117/K120/K125), cluster 2 (cl2: K182/K183/K185) and/or cluster 3 (cl3: K226/K287/K289/K310) (Figure 2a) conserve 70–85% of E2F1 transcriptional activity as compared with the wt protein (Figure 3a). Remarkably, additional mutations at residues K161 and K164 markedly disrupted the E2F1 transcriptional activity (Figure 3a). As observed above, the constructs that contain the K161R/K164R mutation are strongly expressed as compared with their control counterparts (Figure 3a). Whereas E2F1 constructs that contain no (K0), only one (K89) or only 2 (K89 and K137) lysine residues display a very weak transcriptional activity, restoration of K161 and K164 was sufficient to restore up to 75% of the E2F1 transcriptional activity (Figure 3b), highlighting the importance of these two residues. To further study the role of these residues and because HeLa cells express E6 and E7 proteins from human papillomaviruses that could affect the activity of E2F1, we used U2OS cells for functional analysis. In line with our previous results (Figures 3a and b), mutation of K161 and K164 to arginine was sufficient to completely abolish E2F1 transcriptional activity as monitored with *CCNE*, *TP73* and *APAF1* mRNA expression levels (Figure 3d). Moreover, the K161R/K164R mutant was unable to bind the corresponding gene promoters (Figure 3e), although it was still found to be able to bind to DP1, a co-factor that is required for the binding of E2F1 to the DNA (Supplementary Figure 2). We next expressed E2F1 wild-type or the K161R/K164R double mutant in U2OS cells and measured cell proliferation using a crystal violet staining (Figure 3f). E2F1 overexpression was found to significantly slow down cell proliferation, whereas the mutant did not. Overall, our data suggest that K63 ubiquitination on cluster lysine residues 161/164 may be required for E2F1-mediated gene transcriptional regulation.

### DNA damage induces cIAP1-dependent accumulation of K63-ubiquitinated E2F1

**Figure 4:**
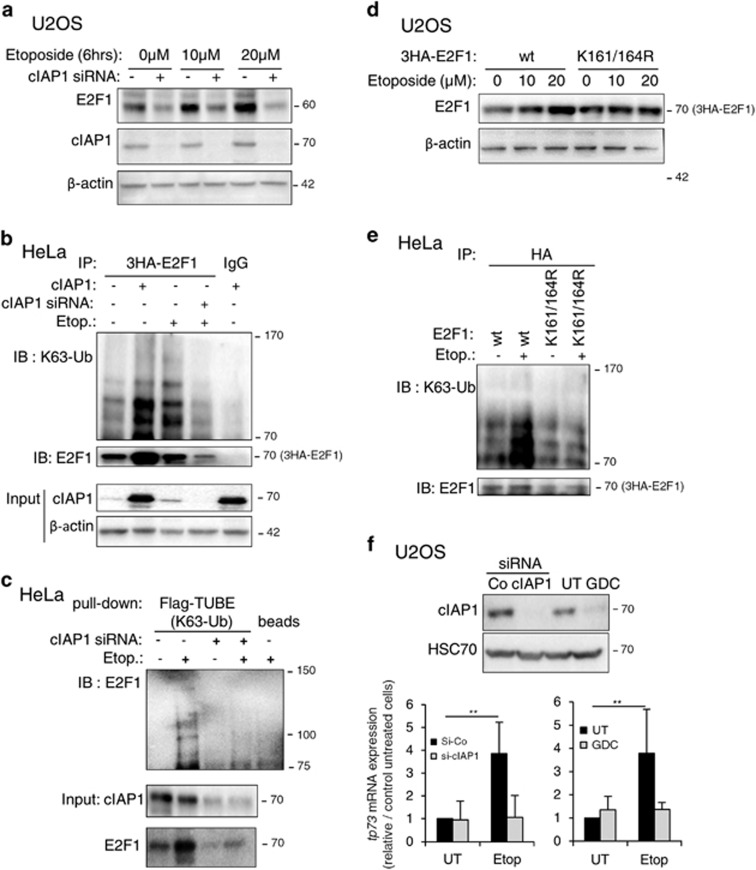
cIAP1 is required for DNA damage-induced stabilization of E2F1. (**a**) Western blot analysis of E2F1 and cIAP1 in U2OS cells transfected with control or cIAP1-siRNA and treated with indicated concentration of etoposide for 6 h. *β*-Actin was used as loading control. (**b**) Ubiquitination profile of E2F1 in HeLa cells transfected with cIAP1 siRNA or cIAP1-encoding construct and with 3HA-E2F1 and His-tagged ubiquitin wt. When indicated, cells were treated for 6 h with 10*μ*M etoposide. E2F1 was IP using anti-HA antibody and ubiquitin revealed using K63-specific ubiquitin chain antibody (K63-Ub). The expression of the transgenes and the efficiency of siRNA were checked by a western blot analysis (input). *β*-Actin was used as loading control. (**c**) Ubiquitination assay performed in HeLa cells transfected with control or cIAP1 siRNA and with 3HA-E2F1 and His-tagged ubiquitin wt. When indicated, cells were treated for 6 h with 10*μ*M etoposide. Ubiquitinated proteins were pulled-down by using K63 specific TUBEs and ubiquitinated E2F1 is revealed by using E2F1 antibody. (**d**) Western blot analysis of E2F1 in U2OS cells transfected with 3HA-E2F1 or 3HA-E2F1-K161/164R mutant and treated with indicated concentration of etoposide for 6 h. *β*-Actin was used as loading control. (**e**) Ubiquitination of E2F1 in HeLa cells transfected with pCMV-3HA-E2F1 or pCMV-3HA-E2F1 K161/164R, and with His-tagged ubiquitin wt, and then treated for 6 h with 10 *μ*M etoposide. E2F1 was IP using anti-HA antibody and ubiquitin revealed using K63-specific ubiquitin chain antibody (K63-Ub). The level of expression of E2F1 constructs has been adjusted in order to get equivalent ubiquitin level in both untreated samples. (**f**) U2OS cells were transfected with cIAP1 siRNA or treated with 17 nM GDC-0152 for 1 h, and then treated with 10 *μ*M etoposide for 48 h. *tp73* mRNA expression was measured by RT-qPCR. UT: untreated cells. Results were normalized to *hprt* mRNA and expressed relatively to control untreated cells. Mean±S.D. of three independent experiments

We next evaluated the role of cIAP1 and ubiquitination of E2F1 in DNA damage-induced stabilization of the protein, which is illustrated here in U2OS cells treated with increasing concentrations of the topoisomerase inhibitor etoposide (Figure 4). Depletion of cIAP1 induced both a decrease in basal E2F1 expression levels and impaired etoposide-mediated E2F1 upregulation (Figure 4a). Etoposide treatment induced the accumulation of K63-ubiquitinated E2F1 proteins with an ubiquitination profile equivalent to the one observed after cIAP1 overexpression, an effect prevented by cIAP1 silencing (Figures 4b and c). Contrary to wt E2F1, the K161R/K164R double mutant failed to undergo stabilization (Figure 4d) and K63 ubiquitination (Figure 4e) in response to etoposide treatment. Consistently, depletion of cIAP1 by using either a siRNA or GDC-0152, an IAP antagonist known to induce a very quick degradation of cIAP1,^18^ prevented etoposide from inducing the expression of the E2F1 target, DNA damage-responsive gene *tp73* (Figure 4f).

### The importance of arginine methylation in cIAP1-mediated accumulation of poly-ubiquitinated E2F1

**Figure 5:**
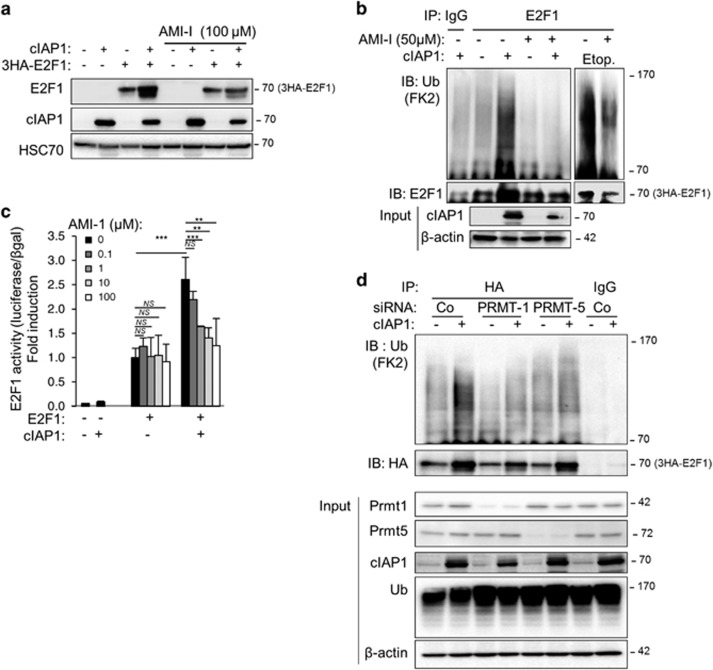
PRMT-mediated arginine methylation of E2F1 is required for regulation of E2F1 by cIAP1. (**a**) Western blot analysis of E2F1 and cIAP1 in HeLa cells transfected with 3HA-E2F1 and cIAP1-encoding constructs, and treated for 24 h with 100 *μ*M PRMT inhibitor AMI-1. HSC70 was used as loading control. (**b**) Ubiquitination of E2F1 in HeLa transfected with pCMV-3HA-E2F1, pCI-cIAP1, and with His-tagged ubiquitin wt, and then treated for 16 h with 50 *μ*M AMI-1±10 *μ*M etoposide for 6 h. E2F1 was IP using anti-E2F1 antibody and ubiquitin revealed using pan anti-ubiquitin antibody (FK2). The expression of the transgene is checked by western blot. *β*-Actin was used as loading control. (**c**) E2F1 transcriptional activity as measured by using a gene reporter luciferase assay performed in Hela cells transfected as in **a** in the presence of CCNE promoter-Firefly luciferase reporter plasmid and treated with increasing concentration of AMI-1 for 24 h. Results are expressed as average±S.D. from at least three experiments. Statistical analysis performed using Student’s *t-*test. ***P<0.001, **0.001<*P*<0.01, NS *P*>0.1. (**d**) Ubiquitination profile of E2F1 in HeLa transfected with Control (Co), PRMT-1 or PRMT-5-siRNA and pCMV-3HA-E2F1, pCI-cIAP1, and with His-tagged ubiquitin wt. E2F1 was IP using anti-HA antibody and ubiquitin revealed using pan anti-ubiquitin antibody (FK2). The expression of the transgene and the efficiency of siRNA were checked by western blot analysis. *β*-Actin was used as loading control

The DNA damage-mediated E2F1 protein stability and activity is regulated by the arginine methyl-transferase PRMT-1.^19^ Interestingly, inhibition of PRMT-1 with its specific inhibitor AMI-1 impaired the cIAP1-mediated E2F1 upregulation (Figure 5a) and poly-ubiquitination (Figure 5b), as well as E2F1 transcriptional activity, in a dose-dependent manner (Figure 5c). Albeit to lower extend, AMI-1 also decreased etoposide-induced ubiquitination of E2F1 (Figure 5b). Accordingly, silencing of PRMT-1 partially prevented cIAP1-mediated E2F1 poly-ubiquitination (Figure 5d). Silencing of PRMT-5, another arginine methyl-transferase that promotes arginine methylation in cycling cells,^20^ also inhibited this event (Figure 5d) suggesting that cIAP1-mediated stabilization is likely to occur during the cell cycle.

### cIAP1-dependent K63 ubiquitination of E2F1 is cell cycle regulated

**Figure 6:**
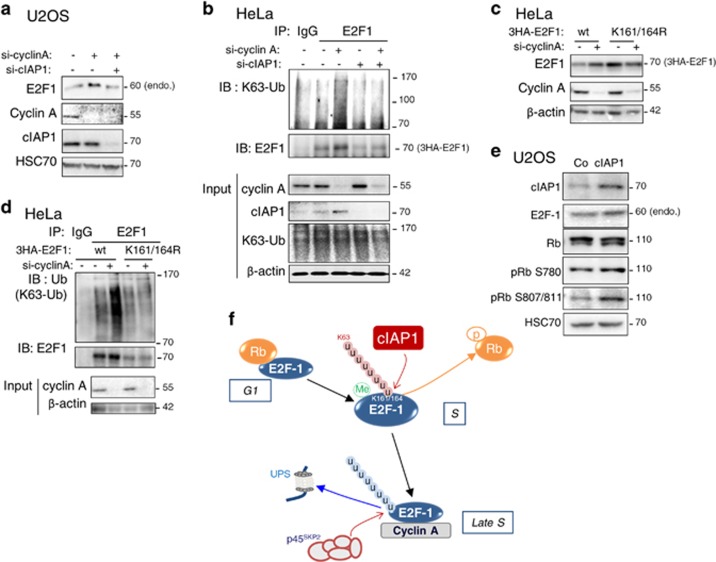
K63 ubiquitination of E2F1 is cell cycle regulated. (**a**) Western blot analysis of E2F1, cyclin A and cIAP1 in U2OS cells transfected with cyclin A siRNA±cIAP1 siRNA. HSC70 was used as loading control. (**b**) Ubiquitination profile of E2F1 in HeLa cells co-transfected with cyclin A siRNA±cIAP1 siRNA, then 24 h later with pCMV-3HA-E2F1 and His-tagged ubiquitin wt. E2F1 was IP using anti-E2F1 antibody and ubiquitin revealed using K63-specific ubiquitin chain antibody (K63-Ub). The expression of the transgenes was checked by a western blot analysis. *β*-Actin was used as loading control. (**c**) Western blot analysis of the expression of 3HA-E2F1 wt or K161/164 R mutant and cyclin A in HeLa cells transfected with cyclin A siRNA. *β*-Actin was used as loading control. (**d**) Ubiquitination profile of E2F1 in HeLa cells co-transfected with cyclin A siRNA, then 24 h later with pCMV-3HA-E2F1 or the K161/164 R mutant and His-tagged K63-only ubiquitin. E2F1 was IP using anti-E2F1 antibody and ubiquitin revealed using K63-specific ubiquitin chain antibody (K63-Ub). The expression of the transgenes was checked by a western blot analysis. *β*-Actin was used as loading control. The level of expression of E2F1 constructs has been adjusted in order to get equivalent ubiquitin level in both samples transfected with control siRNA. (**e**) Western blot analysis of cIAP1 E2F1, cyclin E, Rb, phospho (S780, S807/811) Rb in U2OS cells transfected with cIAP1-encoding construct. HSC70 was used as loading control. (**f**) Schematic representation of the regulation of E2F1. In the G1 phase of cell cycle, E2F1 is complexed to Rb. In the S phase, E2F1 is first methylated on arginine residue par PRMT, then K63-ubiquitinated on K161/164 in a cIAP1-dependent manner, leading to stabilization and activation of the protein. In the late S, cyclin A inhibits K63 ubiquitination of E2F1, binds to and phosphorylates E2F1 and promotes UPS-mediated degradation

The expression and the activity of E2F1 peak in early S phase of cell cycle, and at the end of the S phase, the cyclin A/cdk2 complex binds to and phosphorylates E2F1, leading to its inactivation and its subsequent degradation. We next assessed the role of cIAP1 and poly-ubiquitination of E2F1 in this regulatory process. A downregulation of cyclin A, which blocks cell cycle progression in late S-G2 (Supplementary Figure 3A) and freezes E2F1 in its S phase, induced an increase in the expression of active E2F1 in both U2OS and HeLa cells (Figures 6a and c) and revealed a K63 ubiquitination of the transcription factor (Figure 6b). These two events were completely abolished by cIAP1 depletion using siRNAs (Figures 6a and b). Consistent with the prominent role played by the lysines 161 and 164, the K161/164R E2F1 mutant resisted to cyclin A siRNA-mediated E2F1 accumulation (Figure 6c) and K63 ubiquination (Figure 6d).

In the G1 phase, E2F1 is maintained in an inactive state because of its binding to Rb and phosphorylation of Rb is sufficient to release E2F1, allowing its activation and G1/S phase transition. Overexpression of Rb in HeLa cells, which are Rb deficient, did not affect the ubiquitination status of E2F1 (Supplementary Figure 3B) and downregulation of Rb in U2OS cells, which express wild-type Rb, slightly decreased E2F1 ubiquitination (Supplementary Figure 3C), which is consistent with a decreased S phase (Supplementary Figure 3A). Interestingly, overexpression of cIAP1 in U2OS cells increased the phosphorylation of Rb (Figure 6e). Moreover, an *in vitro* competition experiment indicated that cIAP1 is able to compete with Rb for E2F1 binding (Supplementary Figure 3D), suggesting that cIAP1 may be likely to compete with Rb for E2F1 binding during the cell cycle and thus to promote E2F1 activation (Figure 6f).

## Discussion

The present report highlights a novel level of regulation of the stability and activity of E2F1 protein by a non-degradative ubiquitination process. The turnover of E2F1 is central to the regulation of its transcriptional activity. E2F1 expression peaks at the end of G1 phase of the cell cycle to promote the activation of genes required for the G1–S phase transition and the initiation of DNA synthesis. The expression of E2F1 also considerably rises in response to DNA damage, contributing to the apoptotic response to such damage. Genotoxic stress-induced stabilization of E2F1 was found to involve multiple interconnected post-translational modifications, that is, double-strand DNA breaks promote E2F1 phosphorylation on serine residues 31^21^ and 364,^22^ its acetylation on lysine cluster 117, 120 and 125,^23, 24^ its methylation at Arginine 109,^19^ its demethylation on lysine 185^25^ and arginine 111/113 cluster^19^ and its deNEDDylation.^26^ DNA damage was also found to promote E2F1 ubiquitination,^27^ but, so far, the E3-ubiquitin ligase involved remains unknown. Here, we demonstrate an accumulation of E2F1 K63-ubiquitinated on the lysines 161/164 cluster in cells treated with etoposide, as well as in those locked in the S phase, which is likely to involve the E3-ubiquitin ligase cIAP1.

Although others IAPs including cIAP2 and XIAP also behave as E3-ubiquitin ligases, only cIAP1 stimulates E2F1 activity.^12^ cIAP1, as a dimer,^28, 29^ is able to promote the conjugation of K11, K48 and K63 ubiquitin chains onto protein substrates. cIAP1-mediated K11 and K63 ubiquitination was found to be involved in several ubiquitin-dependent cell signaling events,^11^ for example, in the regulation of NF-*κ*B-activating and TNFR signaling pathways. More specifically, cIAP1-driven K11 and K63 ubiquitination of the kinase RIP1 is known to favors the assembly of a cell signaling protein platform that leads to NF-*κ*B activation, whereas preventing the formation of a RIP1-containing complex that induces cell death.^30, 31, 32, 33^ A role of cIAP1 in transcription regulation has also been described, for example, cIAP1 promotes K48-linked ubiquitin chain conjugation and degradation of the myc antagonist Max-dimerization protein-1 (Mad-1),^34^ as well as the transcription factor CHOP (C/EBP homolog protein).^35^ Similar functions by other IAPs include the ability of XIAP to favor Wnt-dependent transcription program through mediating the mono-ubiquitination of Groucho (Gro)/TLE transcription co-repressor^36^ and the K63 ubiquitination of interferon-regulatory factor 1 by cIAP2.^37^

cIAP1 is able to directly induce the ubiquitination of E2F1 and to promote the accumulation of E2F1 modified with K63-linkage ubiquitin chains on lysine residues 161 and 164. Additional experiments will be required to demonstrate that cIAP1 is indeed the E3-ubiquitine ligase responsible for the K63 ubiquitination of E2F1 in S phase of cell cycle or in response to etoposide. However, as E2F1 accumulation in such situations also involves ubiquitination of lysine residues 161 and 164, cIAP1 is likely to be one of the E3-ligases responsible for these modifications. A K63 ubiquitination of E2F1 has recently been observed but the consequences were not clearly defined.^9, 10^ K63-linked ubiquitin chains are the second most abundant ubiquitin signal in mammalian cells. K63 ubiquitination of proteins is involved in DNA repair, kinase activation, membrane protein internalization, intracellular vesicular trafficking and signaling protein complex assembly.^5^ Our data identified a function for K63 ubiquitination in cell cycle regulation through E2F1 protein stabilization. K63-poly-ubiquitination may counteract the binding of other protein modifiers involved in protein degradation or favor the recruitment of protein chaperones. Accordingly, the mutation of lysine residues 161 and 164 was shown to strongly increase E2F1 NEDDylation.^38^ Stabilization of E2F1 requires PRMT-mediated arginine methylation. Whereas PRMT-1 is important for E2F1 stabilization upon DNA damage,^19^ PRMT-5 promotes arginine methylation in cycling cells.^20^ Here, we show that a downregulation or an inhibition of either PRMT-1 or PRMT-5 decreases the cIAP1-mediated E2F1 regulation, demonstrating the importance of a prior arginine methylation step.

Two main checkpoints control E2F1 activity during the S phase. The first one involves Rb that associates with and represses E2F1 before S phase entry. Rb overexpression does not affect K63 ubiquitination of E2F1. Our results suggest that cIAP1 is able to compete with Rb for E2F1 binding and that cIAP1 overexpression is associated with an increases in Rb phosphorylation, indicating that cIAP1 is likely to favor Rb-E2F1 dissociation to promote E2F1 activation and S phase initiation (Figure 6f). The second checkpoint is at the end of the S phase, when cyclin A:Cdk2 complex binds to and phosphorylates E2F1, leading to the protein inactivation and subsequent degradation by skp2-dependent UPS. We identified an additional level of regulation of this checkpoint by demonstrating that cyclin A affects the K63 ubiquitination of E2F1 (Figure 6f).

The cluster of lysine 161 and 164 residues has been shown to be a target of E2F1 ubiquitination. In addition to completely abolishing the S phase-dependent and the DNA damage-induced accumulation of E2F1, the mutation of K161/K164 cluster also radically impairs E2F1 transcriptional activity, that is, its capacity to bind *CCNE*, *TP73* and *APAF1* gene promoters. Consistent with these observations, downregulation of cIAP1 was found to block the etoposide-induced expression of the E2F1 transcriptional target *TP73*. These results are consistent with our previous work demonstrating that silencing of cIAP1 inhibits E2F1-dependent expression of *CCNE* and *CCNA* genes in cycling cells through inhibition of E2F1 recruitment on the corresponding gene promoters.^12^ We also showed that cIAP1, which interacts with E2F1 in all phases of the cell cycles, is recruited, along with E2F1, onto E2F-binding sites of *CCNE* and *CCNA* promoters during the S phase, when E2F1 activity is at its maximum.^12^ Our current results suggest that E2F1 ubiquitination is key in inducing E2F1 interaction with these target gene promoters, possibly through recruitment of transcriptional co-regulators containing ubiquitin binding modules^39^ to form transcriptional complexes. The deubiquitinases (DUB) POH1 and UCH37, both described as a K63-DUB, were found to regulate E2F1 stability or activity, although with opposite effects.^9, 10^ POH1-mediated deubiquitination appeared to lead to E2F1 stabilization,^10^ whereas UCH37-mediated deubiquitination enhanced E2F1 transcriptional activity.^9^ The role of each of these two DUB in removing DNA damage and S phase-associated K63 ubiquitin chains from E2F1 merits further investigations. Mutation of E2F1 K161/164 does not completely abolish E2F1 K63 ubiquitination profile (Figure 2d) meaning that other lysine residues may be conjugated with K63 ubiquitin chains.

In summary, we demonstrated that cIAP1 as an E3-ubiquitin ligase is able to promote the accumulation of E2F1 K63-ubiquinated on the clustered lysine residues 161 and 164. Such cIAP1-dependent E2F1 modifications was observed both in the S phase of the cell cycle and in response to DNA damage, two situations in which E2F1 transcriptional activity is optimal, suggesting a general mechanism of E2F1 activity regulation. Our findings suggest that IAP-targeting molecules could be tested for their ability to overcome E2F1 activity in tumor cells, for example, in an Rb-compromised setting.

## Materials and methods

### Cell culture and treatment

Human cervical carcinoma cell line (HeLa), human osteosarcoma epithelial cell line (U20S) and mouse embryonic immortalized (SV40) fibroblasts (MEF) cIAP1^−/−^/cIAP2^−/−^ (J Silke, Melbourne, Australia), were grown in Dulbecco’s modified Eagle‘s medium (Dominique Dutscher, Brumath, France) or RPMI 1640 (Roswell Park Memorial Institute, Dominique Dutscher) containing 10% fetal bovine serum (Dominique Dutscher) at 37 °C in a 5% CO_2_ atmosphere and 95% humidity. Cells were treated with 10 or 20 *μ*M etoposide (Sigma-Aldrich, Lyon, France) for 6 h, 100 *μ*g/ml cycloheximide (Sigma-Aldrich), 10–35 *μ*M PYR-41 (Calbiochem, Merck Millipore, Saint-Quentin en Yvelines, France) for 24 h, 0.1–100 *μ*M AMI-I (Enzo Life Sciences, Villeurbanne, France) for 24 h, or 1 *μ*M GDC-0152 (Selleckmed, Euromedex, Souffelweyersheim, France). MG132 (Sigma-Aldrich,) was used at 1 *μ*M or 5 *μ*M overnight.

#### Plasmid constructs and siRNA transfections

Plasmids used were pCI empty vector, pCI-cIAP1, pCI-cIAP1-H588A, pCMV-*β*Gal, pCMV-HA-DP1, pGL3 human CCNE promoter;^12^ pcDNA3.1-6His-Ub wt and 6His-Ub-K63-only mutants in which all K have been mutated into R except the K63; pCMV-HA-E2F1 wt and pCMV-HA-E2F1 K117/120/125R,^17^ pCMV-3HA-E2F1 and pCMV-3HA-E2F1-K185R;^25^ pCMV-Flag-E2F1 wt and mutants that contain no lysine (K0) or only K89 (K89 only), K89 and K137 (K89, 137), K89, K137, K161 and K164 (K89, 137, 161, 164) or in which K117/120/125) (cluster 1), K182/183/185 (cluster 2) and/or K226/287/289/310 (cluster 3) have been mutated into R.^26^ The pCI-cIAP1-F616A mutant was obtained by site-directed mutagenesis from pCI-cIAP1 with GENEART Site-Directed Mutagenesis system (Invitrogen, ThermoFisher Scientific, Villebon-sur-Yvette, France) by using the following primers: forward (5′-CAAGGGTACTGTTCGTACAGCTCTCTCATAATCGACCCG-3′), reverse (5′-CGGGTCGATTATGAGAGAGCTGTACGAACAGTACCCTTG-3′). The pCMV-3HA-E2F1 mutants (K89R, K137R, K161/164R, K181/183R, K266R, K287/289R, K310R) were obtained by site-directed mutagenesis from pCMV-3HA-E2F1 (GenScript, Piscataway, NJ, USA). Plasmids constructs were transiently transfected using JetPEI (Polyplus transfection, Ozyme, Montigny-le-Bretonneux, France). Lipofectamine RNAimax reagent (ThermoFisher Scientific, Villebon-sur-Yvette, France) was used to transfect siRNAs. RNA oligonucleotides are designed and purchased from Qiagen (Qiagen France SAS, Courtaboeuf, France) for cIAP1, Rb, cyclin A and control siRNA or Ambion (Thermo Fisher Scientific) for PRMT-1 and PRMT-5 siRNA.

#### Gene reporter assay

The transactivation activity of E2F1 was analyzed as previously described.^12^

#### Cellular extracts and western blot analysis

Cells were lysed in RIPA buffer (NaCl 150 mM, NP40 1%, deoxycholate sodium (DOC) 0.5%, SDS 0.1%, Tris pH 7.5 50 mM and protease inhibitor cocktail). Proteins were separated on SDS-PAGE and electro-transferred onto polyvinylidene difluoride membranes (GE Healthcare, Dominique Dutscher, Brumath, France). Blots were probed with the following antibodies: a rabbit polyclonal anti-E2F1 (C20), a mouse monoclonal anti-HSC70 and a mouse monoclonal anti-PRMT-5 (A-11) from Santa Cruz Biotechnology (Santa Cruz Biotechnology, Clinisciences, Naterre, France), a goat polyclonal anti-cIAP1 from R&D Systems (Minneapolis, MN, USA) (AF8181); a rabbit polyclonal anti-PRMT-1 from Upstate, Upstate Biotechnology (Merck Millipore), a mouse anti-*β*-actin (AL978) and a mouse anti-*α*-tubulin (clone AA13) from Sigma-Aldrich (Lyon, France), a monoclonal FK2-HRP-conjugated anti-ubiquitin (mono and poly-ubiquitin chains), a monoclonal HRP-conjugated anti-K63 linkage-specific ubiquitin (HWA4C4) from Enzo Life Sciences, a mouse monoclonal anti-HA.11 (Biolegend, San Diego, CA, USA), a mouse anti-cyclin A (BD Biosciences Grenoble, France), a rabbit anti-Rb and a rabbit anti-phospho-Rb (Ser780 or Ser807/811) (Cell Signaling Technology, Ozyme, Saint-Quentin en Yvelines, France). Detection was performed using peroxidase-conjugated secondary antibodies and chemiluminescence detection kit (ClarityTM western ECL substrate, Bio-Rad, Marnes-la-Coquette,France).

### Ubiquitinylation assay

For the *in vitro* ubiquitination assay, GST-E2F1 fusion protein was produced in *Escherichia coli* by using the pGEX-E2F1 construct, immobilized on gluthatione sepharose beads (GE Healthcare) and incubated Sigma at 37 °C for 1 h with recombinant cIAP1 (R&D System), 100 nM of recombinant human E1 ubiquitin-activating enzyme UBE1 (Boston Biochem, Cambridge, MA, USA), 1.5 *μ*g of recombinant human E2 enzyme Ubc H5a/UBE2D1 (Boston Biochem), 0.2 mM of recombinant human ubiquitin (R&D Systems) in a buffer containing 75 mM Tris pH 8.2 mM DTT, 5 mM de MgCl_2_, 4 mM ATP. Gluthatione sepharose beads were then washed and proteins were eluted in Leammli buffer. E2F1 ubiquitination was revealed using anti-E2F1 antibody immunoblotting.

For the ubiquitination assays, HeLa cells were transfected with 5 *μ*g of each plasmid encoding ubiquitin wt or K63-only mutant, HA-E2F1 wt or mutants, cIAP1 wt or mutants. Cells were treated overnight with 1 *μ*M MG132 (Sigma) and with the non-selective DUB inhibitors PR619 10 (LifeSensors, Tebu-bio SAS, France) before cell lysis. Ubiquitinated proteins were pulled-down following the manufacturer instructions by using Flag-conjugated anti-K63 TUBEs, Agarose-TUBEs or GST-TUBEs (LifeSensors) and revealed by using anti-E2F1 antibody. For the analysis of ubiquitination profile of E2F1, 3HA-E2F1 was immunoprecipitated (IP) with anti-E2F1 or anti-HA antibody and revealed by using monoclonal FK2-HRP-conjugated anti-ubiquitin (mono and poly-ubiquitin chains) or a monoclonal HRP-conjugated anti-K63-linkage specific ubiquitin (HWA4C4) (Enzo Life Sciences).

#### Immunoprecipitation

Cells (10^6^ per conditions) were transfected with plasmids encoding E2F1 wt or K161/164 R mutant, cIAP1 wt or DP1. Forty-eight hours after transfection, cells were washed with PBS and lysed in IP Lysis buffer (150 mM de NaCl, 50 mM Tris HCl pH 7,4, 20 mM EDTA, 0,5% NP40, 1 mM DTT, 5 mM *N*-ethylmaleimide (Sigma-Aldrich) and protease inhibitors) for 30 min at 4 °C. Lysate supernatants were precleaned by using 20 *μ*l of Protein A/G+ Agarose beads (Sigma) and incubated (overnight, 4 °C) with rabbit polyclonal anti-E2F1 (C20) antibody (Santa Cruz Biotechnology), mouse IgG1 purified anti-HA.11 (Biolegend), goat polyclonal anti-cIAP1 (R&D Systems) or Igg anti-mouse or anti-rabbit antibody. Beads were then washed in IP lysis buffer and denaturated in Laemmli buffer before immunoblot analysis.

#### GST-pull down analysis

GST-pull down analysis was performed as previously described.^40^

#### RNA Purification, reverse transcription, real-time PCR (qPCR)

Total RNAs were isolated using TRIzol Reagent (Invitrogen, ThermoFisher Scientific). RNA quality was ascertained using a nanospectrophotometer (Nanodrop 2000, Thermo Scientific, Waltham, MA, USA). Total RNA (1 *μ*g) was reverse- transcribed using iScript cDNA synthesis kit (Bio-Rad) according to manufacturer’s instructions. Quantitative real-time PCR was performed with 7500 Fast thermocycler or StepOne plus system (Applied Biosystem, ThermoFisher Scientific) using SYBR Green detection protocol and the following primers: TP73 promoter forward: 5′-TGAGCCATGAAGATGTGCGAG-3′, reverse: 5′-GCTGCTTATGGTCTGATGCTTATG-3′ APAF1 promoter forward: 5′-GGAGACCCAGGACGACAA-3′ reverse: 5′-CAGTGAAGCAACGAGGATGC-3′ CCNE promoter forward: 5′-CCATCGGCCATCTTCCTGGCTC-3′, reverse: 5′-TCAGGCCGCGGGCCCAGTA-3′ CCNE forward: 5′-GCCAGCCTTGGGACAATAATG-3′, reverse: 5′-CTTGCACGTTGAGTTTGGGT-3′; P73 forward: 5′-GACGAGGACACGTACTACCTT-3′, reverse: 5′-CTGCCGATAGGAGTCCACCA-3′; APAF1 forward: 5′-TCACTGCAGATTTTCACCAGA-3′, reverse: 5′-CCTCTCATTTGCTGATGTCG-3′; HPRT forward: 5′-GGACAGGACTGAACGTCTTGC-3′, reverse: 5′-CTTGAGCACACAGAGGGCTACA-3′.

#### Chromatin IP

Hela cells (1.5 × 10^6^ per conditions) were transfected with 5 *μ*g of pCMV-3HA-E2F1 wt or K161/164 R mutant encoding vectors and 10 *μ*g of PCI control vector, then protein/DNA complexes were cross-linked with formaldehyde 1%. Chromatid was isolated and digested with micrococcal nuclease. Ten percent of lysate containing the digested chromatid was conserved for input. The remaining lysate is diluted and IP using 5 *μ*g of rabbit anti-E2F1 (C20) or rabbit control Igg (Santa Cruz Biotechnology) overnight. Elution and DNA recovery were performed according to recommendation of PIERCE Agarose Chip Kit (Thermo Scientific). Specific chromatin associated with interest protein was analyzed by qPCR as described above.

#### Cell proliferation

U2OS cells were transfected with pCMV-3HA-E2F1 wt or K161/164 R mutant encoding vectors. Twenty-four hours later, transfected 20 000 U2OS cells were implanted in six-well plates for 6 days. Six days later, cells were fixed by ethanol (100%) and stained with crystal violet. Crystal violet staining was eluted with acetic acid (33%) and the optical density at 620nm was measured with a plate reader (UVM 340, Biochrom, Cambourne, UK).

#### Cell cycle analysis

U2OS cells (0.65 × 10^5^ per conditions) were transfected with 20 *μ*M of control or cyclin A-siRNA, or 50 *μ*M Rb-siRNA. Cell cycle is evaluated by using the BrdU Flow kit (BD Bioscience) 48 h after transfection according to the manufacturer instruction and cell cycle repartition was assessed by BD FACS Canto II flow cytometer using Flowing Software 2 (Call imaging Core, Turku Centre for Biotechnology, Turku, Finland).

#### Statistical analysis

Student’s *t*-test or the Mann–Whitney Wilcoxon test was used for statistical analysis.
