# Correction: DNA damage and S phase-dependent E2F1 stabilization requires the cIAP1 E3-ubiquitin ligase and is associated with K63-poly-ubiquitination on lysine 161/164 residues

**DOI:** 10.1038/s41419-018-0822-4

**Published:** 2018-07-16

**Authors:** Valérie Glorian, Jennifer Allègre, Jean Berthelet, Baptiste Dumetier, Pierre-Marie Boutanquoi, Nathalie Droin, Cémile Kayaci, Jessy Cartier, Simon Gemble, Guillaume Marcion, Daniel Gonzalez, Romain Boidot, Carmen Garrido, Olivier Micheau, Eric Solary, Laurence Dubrez

**Affiliations:** 1 Université de Bourgogne Franche-Comté, LNC UMR1231, Dijon, France; 2 Institut National de la Santé et de la Recherche Médicale (Inserm), LNC UMR1231, Dijon, France; 3 Inserm U1170, Gustave Roussy, Villejuif, France; 4 Centre Georges-François Leclerc, Dijon, France; 5 Université Paris-Sud, Faculté de Médecine, Le Kremlin Bicêtre, France

**Correction to:** *Cell Death & Disease* **8**, e2816 (2017); 10.1038/cddis.2017.222; published online 25 May 2017.

Following publication of this article, it was noticed that an error during typesetting had led to the author Olivier Micheau being listed incorrectly as Olivier Michaud. The publisher would like to apologise for any inconvenience caused.

