# Supplementary material for: DNA damage and S phase-dependent E2F1 stabilization requires the cIAP1 E3-ubiquitin ligase and is associated with K63-poly-ubiquitination on lysine 161/164 residues
Source: Cell Death Dis. 2017 May 25;8(5):e2816–. doi: 10.1038/cddis.2017.222 (PMC5520736; doi:10.1038/cddis.2017.222)
Supplement: Supplementary Figures [file cddis2017222x1.ppt]

## Slide 1
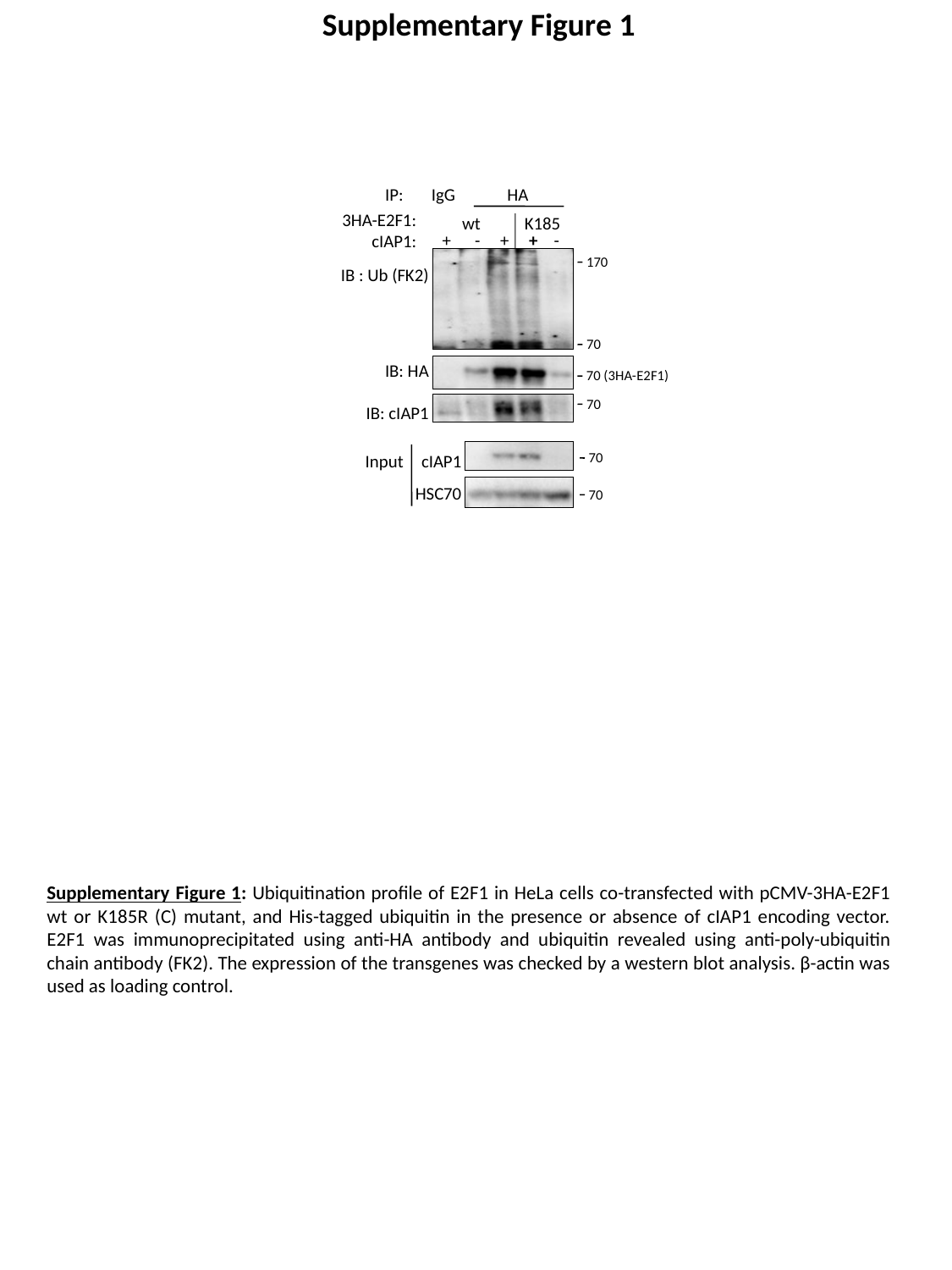

Supplementary Figure 1
IP: IgG HA
3HA-E2F1:
cIAP1:
wt K185
 + - + + -
170
IB : Ub (FK2)
IB: HA
IB: cIAP1
70
70 (3HA-E2F1)
70
Input
 cIAP1
HSC70
70
70
Supplementary Figure 1: Ubiquitination profile of E2F1 in HeLa cells co-transfected with pCMV-3HA-E2F1 wt or K185R (C) mutant, and His-tagged ubiquitin in the presence or absence of cIAP1 encoding vector. E2F1 was immunoprecipitated using anti-HA antibody and ubiquitin revealed using anti-poly-ubiquitin chain antibody (FK2). The expression of the transgenes was checked by a western blot analysis. β-actin was used as loading control.

## Slide 2
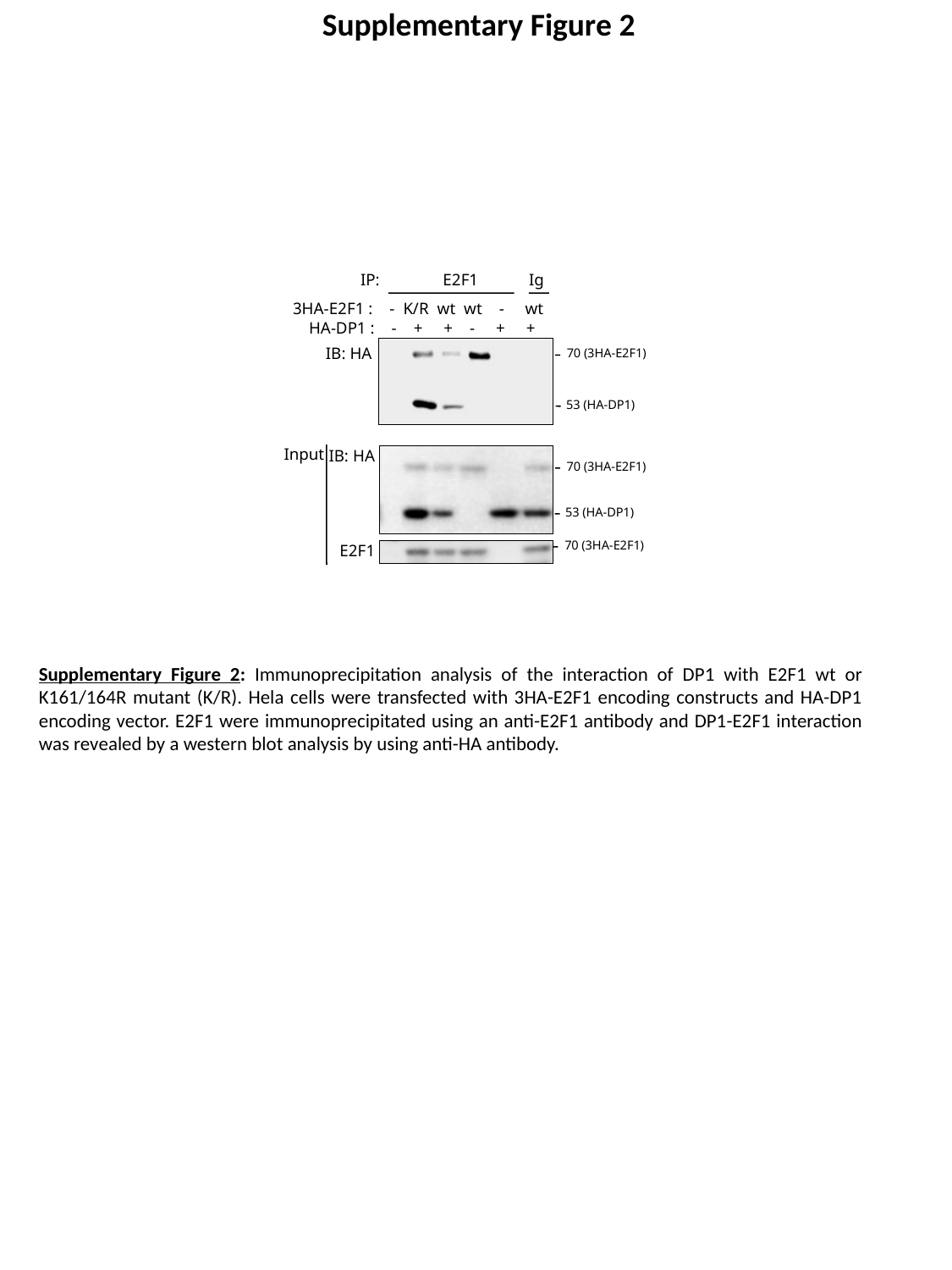

Supplementary Figure 2
IP: E2F1 Ig
3HA-E2F1 : - K/R wt wt - wt
HA-DP1 : - + + - + +
IB: HA
70 (3HA-E2F1)
53 (HA-DP1)
Input
IB: HA
E2F1
70 (3HA-E2F1)
53 (HA-DP1)
70 (3HA-E2F1)
Supplementary Figure 2: Immunoprecipitation analysis of the interaction of DP1 with E2F1 wt or K161/164R mutant (K/R). Hela cells were transfected with 3HA-E2F1 encoding constructs and HA-DP1 encoding vector. E2F1 were immunoprecipitated using an anti-E2F1 antibody and DP1-E2F1 interaction was revealed by a western blot analysis by using anti-HA antibody.

## Slide 3
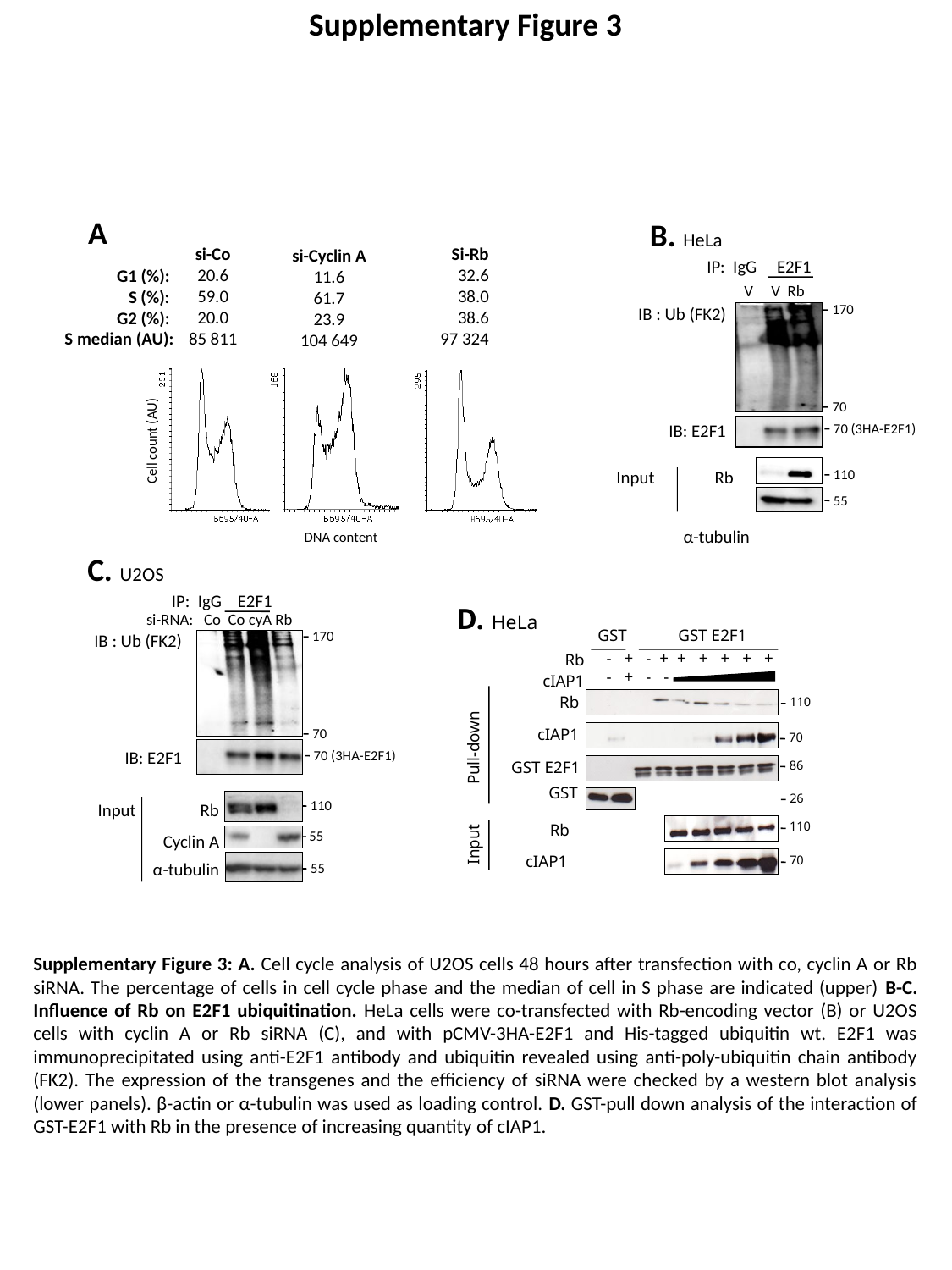

Supplementary Figure 3
A
B. HeLa
IP: IgG E2F1
V V Rb
170
IB : Ub (FK2)
IB: E2F1
70
70 (3HA-E2F1)
Input Rb
α-tubulin
110
55
si-Co
20.6
59.0
20.0
85 811
Si-Rb
32.6
38.0
38.6
97 324
si-Cyclin A
11.6
61.7
23.9
104 649
G1 (%):
S (%):
G2 (%):
S median (AU):
DNA content
Cell count (AU)
C. U2OS
IP: IgG E2F1
 si-RNA: Co Co cyA Rb
170
IB : Ub (FK2)
IB: E2F1
70
70 (3HA-E2F1)
Input Rb
Cyclin A
α-tubulin
110
55
55
D. HeLa
GST
GST E2F1
- + - + + + + + +
- + - -
Rb
cIAP1
Rb
110
cIAP1
70
Pull-down
86
GST E2F1
GST
26
110
Rb
Input
cIAP1
70
Supplementary Figure 3: A. Cell cycle analysis of U2OS cells 48 hours after transfection with co, cyclin A or Rb siRNA. The percentage of cells in cell cycle phase and the median of cell in S phase are indicated (upper) B-C. Influence of Rb on E2F1 ubiquitination. HeLa cells were co-transfected with Rb-encoding vector (B) or U2OS cells with cyclin A or Rb siRNA (C), and with pCMV-3HA-E2F1 and His-tagged ubiquitin wt. E2F1 was immunoprecipitated using anti-E2F1 antibody and ubiquitin revealed using anti-poly-ubiquitin chain antibody (FK2). The expression of the transgenes and the efficiency of siRNA were checked by a western blot analysis (lower panels). β-actin or α-tubulin was used as loading control. D. GST-pull down analysis of the interaction of GST-E2F1 with Rb in the presence of increasing quantity of cIAP1.
